# Diagnosis of Intoxication by the Organophosphate VX: Comparison Between an Electrochemical Sensor and Ellman´s Photometric Method

**DOI:** 10.3390/s8095229

**Published:** 2008-09-01

**Authors:** Miroslav Pohanka, Martina Hrabinova, Kamil Kuca

**Affiliations:** 1 Centre of Advanced Studies and Department of Toxicology, Faculty of Military Health Sciences, University of Defense / Trebesska 1575, 50001 Hradec Kralove, Czech Republic; E-Mails:; hrabinova@pmfhk.cz (M. H.); kucakam@pmfhk.cz (K. K.)

**Keywords:** Organophosphate, carbamate, intoxication, diagnosis, biosensor

## Abstract

An electrochemical sensor is introduced as a tool applicable for diagnosis of intoxication by cholinesterase inhibitors caused by the well-known nerve agent VX. The traditional Ellman method was chosen for comparison with the sensor's analytical parameters. Both methods are based on estimation of blood cholinesterase inhibition as a marker of intoxication. While Ellman's method provided a limit of detection of 5.2×10^-7^ M for blood containing VX, the electrochemical sensor was able to detect 4.0×10^-7^ M. Good correlation between both methods was observed (R = 0.92). The electrochemical sensor could be considered a convenient tool for a fast yet accurate method, easily available for field as well as laboratory use. Time and cost savings are key features of the sensor-based assay.

## 1. Introduction

Organophosphate pesticides and nerve agents represent a wide group of toxic compounds having common ability to bind active serine in acetylcholinesterases (AChEs; EC 3.1.1.7) and butyrylcholinesterases (BChEs; EC 3.1.1.8) in a complex mechanism including oxidative stress [1]. The organophosphate pesticides group is quite extensive [2]. The well-known representatives are e.g. the very toxic paraoxon and less toxic parathion, which spontaneously forms paraoxon under wet conditions or in the body [3]. Some of pesticides, such as trichlorfon (metrifonate), were found useful for treatment of Alzheimer's disease cognitive manifestation [4]. The number of organophosphate nerve agents is not so extensive. Probably the best known are sarin, cyclosarin, soman, tabun, and Vx [5]. Nerve agents are usually named after their discoverer(s). For example, sarin is named after the team of synthetic chemists (Schrader, Ambros, Rudigen and Van der Linde) who first prepared it. Sarin, cyclosarin, coman and tabun are the so called G- agents (after **G**erman scientist Dr. Gerhard Schrader and his team). Another group (VE, VG, VM, VR, VX) are the V (for “Victory”) compounds.

The main toxicological effect of AChE inhibitors is towards central nervous system (CNS) [6]. AChE acts as an important part of nerve synapses where it hydrolyses the neurotransmitter acetylcholine; however, the recent data points to more extensive function in organisms [7]. Serious intoxication leads to a cholinergic crisis affecting the nervous system [8]. The seriousness of intoxication is proportional to the organophosphate intake. Mild forms of intoxication are evident by dilated pupils. Higher intake leads to CNS paralysis [9].

Though many instrumental analytical methods such as mass spectrometry or gas chromatography are usable for organophosphates [10], dipsticks [11, 12] and biosensors [13] are the methods of choice. Biosensors could be based on a simple electrochemical principle being applicable for assay of wide spectrum of AChE inhibitors [14, 15] and pharmacological studies [16].

The correct diagnosis of intoxication is quite difficult, even if the activity of blood AChE and BChE could be measured [17] using routinely available Ellman's reagent [5,5′-dithiobis(2-nitrobenzoic acid)] (indicated in the text by the abbreviation DTBN) which produces a 2-nitro-5-thiobenzoate anion that absorbs strongly at 412 nm [18]. However, the assay has an extremely high background absorbance due to similar absorbance spectrum of hemoglobin. Electrochemical detection is based on a completely different principle: first, the alternative substrate acetylthiocholine is enzymatically hydrolyzed to acetic acid and thiocholine. In the second step, thiocholine is oxidized by the applied voltage instead of reaction with Ellman's reagent needed for the photometrical assay. The present study is focused on diagnosis of organophosphate intoxication using an electrochemical sensor. The proposed sensor is expected to be more useful for routine measurements when compared with more traditional photometrical assay.

## 2. Results and Discussion

Two methods for estimation of intoxication by organophosphates, represented by the well-known nerve agent VX, were compared. Ellman's method was selected as the assay routinely used for this purposes. The proposed use of an electrochemical sensor for diagnosis of intoxication is a novel idea. The scale of intoxication was measured in a similar manner for both methods, using the mathematical formulae expressed as equations 1 (for Ellman's assay) and 2 (for the sensor based assay) (see Experimental). Spiked blood was used for the experiments as it was considered a convenient model independent of possible individual discrepancies between different animals.

**Figure 1.**
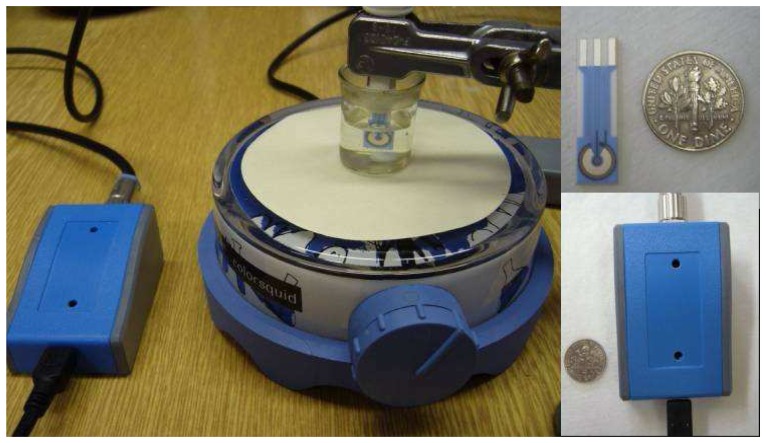
Photograph of the electrochemical device. Complete apparatus with EmStat detector, magnetic stirrer, reaction cell and sensor is presented in the left side. The reaction cell in the photograph was filled with water for better viewing. The screen-printed electrochemical sensor is presented in the right upper photograph. A blow-up of the EmStat detector is presented in the right bottom photograph. A U.S. dime coin (17.91 mm diameter) is shown for photographic scale estimation.

The electrochemical devices used throughout our experiments are commercially available alternatives to the more elaborate ones needed in other assays. The electrochemical detection system used is presented in Figure 1. The device was chosen as a readily available platform for possible widespread application. The screen printed sensors used in the experiments could be washed with phosphate-buffered saline with Tween 20 (PBST) and reused, without an hysteretic influence on the proposed assay due to the stability of noble metal electrodes, although it was noted that the insulating lacquer surface of the electrodes was damaged after approximately thirty measurement cycles, complicating subsequent surface washing.

### 2.1. Measurements on intoxicated blood samples

**Figure 2.**
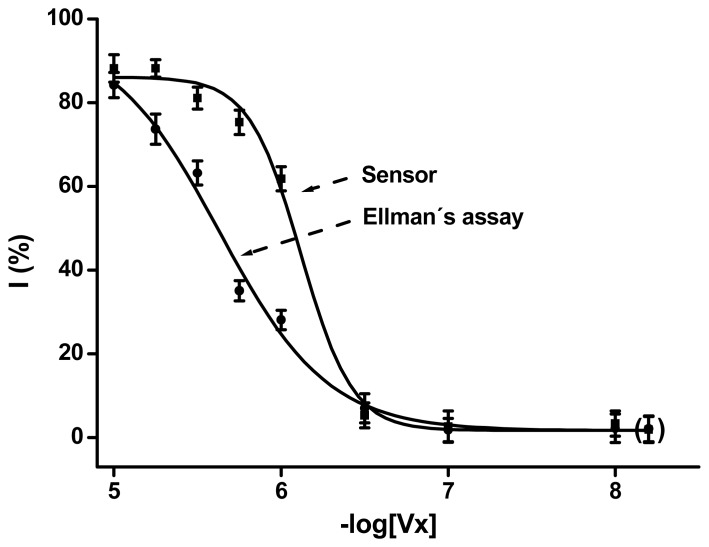
Presentation of calibration curves (percent of inhibition vs. VX concentration) for Ellman's method and the electrochemical sensor. Concentration of VX in blood is expressed as the negative logarithm of molar concentration. Error bars indicate standard deviations (n=4) and points in brackets blank (unspiked blood).

Ellman's method as well as electrochemical sensors were used for diagnosis of intoxication by organophosphates represented by the very toxic VX. The physical principles of both assays are qute different, but the degradation of acetylthiocholine by blood cholinesterases is a common step. The traditional Ellman's method is based on the interaction between the produced thiocholine and DTBN. The method proposed here is based on a completely different principle: electrochemical oxidation of thiocholine. The sensor-based platform is more suitable for routine assays, due to miniaturization and simple mass production,when compared with the photometrical devices needed for Ellman's method. The principles of Ellman's method and the electrochemical sensor are described in the Experimental section. Thiolate anion is the final products of the DTBN and dithiocholine reaction product. Complete calibration curves are shown in Figure 2. Deviations of both methods are of the same level. The achieved limit of detection for the electrochemical sensor was 4.0×10^-7^ M of Vx in blood. The lmit of detection of Ellman's method was only slightly higher: 5.2×10^-7^ M.

### 2.2. Comparison of both methods

**Figure 3.**
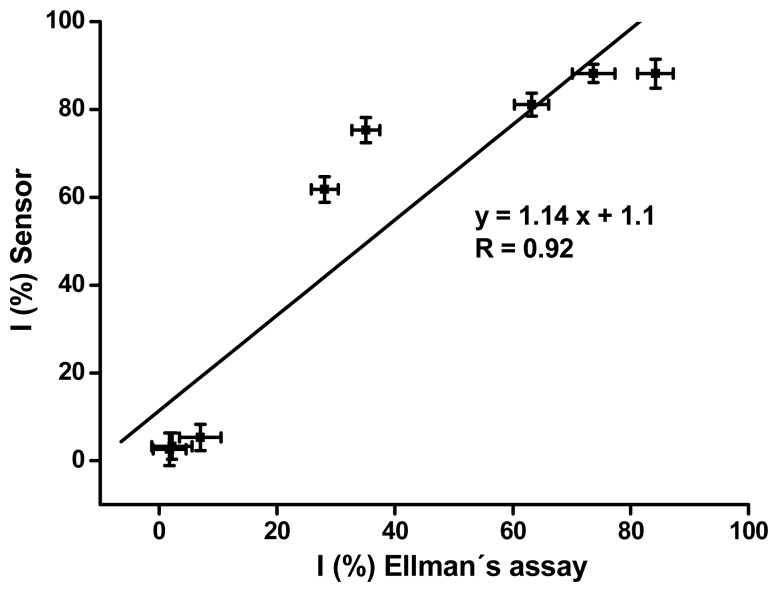
Comparison of electrochemical sensor and Ellman's assay for diagnosis of intoxication by cholinesterase inhibitors, represented by the nerve agent VX. Percent of inhibition was used as final output provided by both methods. Experimental data from Figure 2 were used for graph construction.

Both methods seem to be useful for diagnosis of intoxication by organophosphates using blood cholinesterases as markers. The overall shape presented in Figure 2 and the limits of detection are quite similar. The electrochemical sensor provided only a slightly better limit of detection. Comparison of the achieved percent of inhibition by the sensor vs. Ellman's method is presented in Figure 3. The sensor based assay was more sensitive for inhibited blood approximately in the 10 – 60 % range. The bottom and upper level of inhibition was diagnosed on a similar level for the both methods. The similar level of limit of detection is caused by the similar sensitivity at the lower inhibition level.

### 2.3. Overall impact

The electrochemical sensor was found useful for diagnosis of intoxication by cholinesterase inhibitors represented as a convenient model by pig blood spiked with the nerve agent VX. Though analytical parameters of the presented assay confrm the good performance of the traditional Ellman's method, as well as electrochemical sensor, it seems that the electrochemical sensor is slightly more sensitive. The overall usability of the electrochemical sensor is also an advantage. A simple, convenient electrochemical device for chronoamperometrical assay is readily available considering overall costs. Moreover, costs per assay are also minimal. Only ATChCl is needed as reagent for the assay. The reaction cell and electrodes could be reused many times. The whole electrochemical device is quite miniaturized and completely portable. Moreover, further miniaturization is simple, as may be seen in modern personal glucometers operating on the same transducer principle. The electrochemical sensor is less sensitive to impurities and especially blood clots, a common problem in optical assays of blood samples. For these reasons, it is also favorable for diagnosis of intoxication in dead animals. The power of the electrochemical sensor method is clearly visible from the presented study. Improvements for routine performance can be expected in the future, especially, when apparatus size and costs are considered. The apparatus could be very useful for field applications in order to diagnose intoxicated individuals during asymmetric war events and disasters arising from mishandling of organophosphate or carbamate pesticides. Present practices involve detection of the intoxicant itself. When intoxication appears, the intoxicated individuals are examined in hospitals. A simple detection device such as an electrochemical sensor could be useful in these events in order to perform fast diagnosis. Moreover, s fast field assay could be used for diagnosis of intoxication in animals found in given area as a sign of organophosphates misuse.

## 3. Experimental Section

### 3.1 Blood processing

Fresh pig blood was used for all experiments. Blood was collected and immediately mixed with Heparin Forte (Leciva, Czech Republic). VX (*S*-2-[diisopropylamino]-*O*-ethyl methylphosphonothioate) was kindly provided by the Military Technical Institute (Brno, Czech Republic). Purity was evaluated by acidimetric titration. Blood was mixed with VX up to final concentrations (-log[VX]) of: 0 (control) - 5 – 5.25 – 5.5 – 5.75 – 6 – 6.5 – 7 – 8 and allowed to incubate for 1.5 hour. Blood samples (200 μL) were lysed with 0.02 M TRIS HCl buffer (1.8 mL) with 0.01% Triton X-100 within 10 minutes. The hemolysate was processed immediately because of poor long-term cholinesterase stability under these conditions.

### 3.2 Photometric assay based on Ellman s reagent

A polystyrene cuvette was filled with DTBN (0.4 mg/mL, 0.4 mL), 0.1 M TRIS-HCl (1.3 mL), and hemolyzate in TRIS-HCl and Triton X-100 (100 μL). Reaction was started by immediate injection of 1 mM acetylthiocholine chloride (ATChCl, 0.2 mL). Absorbance against blank (mixture of DTBN, TRIS-HCl, Triton X-100 and hemolyzate) was measured at the beginning and then after one minute using an Helios Alfa device (Thermo Fisher Scientific). The percent of inhibition (*I*) was measured according to the following equation:

(1) $$I=1-\frac{\Delta A_{1,i}-\Delta A_{0,i}}{\Delta A_{1,n}-\Delta A_{0,n}}$$

where Δ*A_1,i_* and Δ*A_0,i_* indicate absorbance at 436 nm provided by VX inhibited blood cholinesterases after one minute of incubation respectively at the experiment beginning (time zero). The symbols in denominator represent non-inhibited blood (intact). The principle behind Ellman's method is shown seen in the following reaction scheme: 
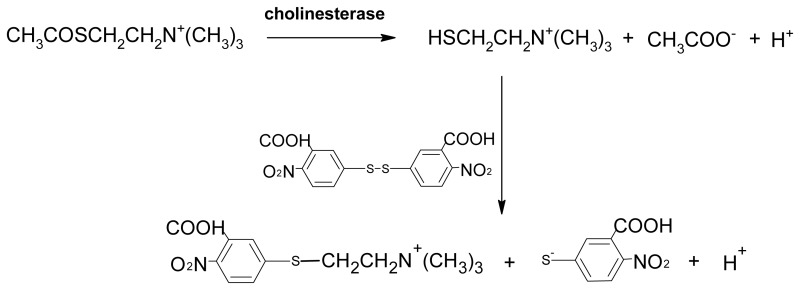


In the first step, acetylthiocholine is split by blood cholinesterase. The liberated thiocholine could interact with DTBN producing a mixed thioester and yellow thiolate anion (absorbing at 436 nm).

### 3.3. Assay based on electrochemical sensor

Electrochemical sensor experiments were performed in a similar way to the Ellman's method ones. Measurements were performed using an EmStat amperometrical device (Houten, Netherlands) and a 3 mL sized reaction cell mixed with magnetic stirrer. The blood lysate (0.5 mL) prepared as mentioned above and 1 mM ATChCl (1.5 mL) was injected into the cell. The screen-printed electrochemical sensor included a platinum working electrode (dot shaped with 1 mm diameter), a Ag/AgCl reference electrode (circle shaped) and a Pt auxiliary electrode (circle shaped). The sensor was immersed into the cell, the applied voltage needed for thiocholine oxidation was set at +410 mV and inhibition was measured in the following way:

(2) $$I=1-\frac{i_{1,i}-i_{0,i}}{i_{1,n}-i_{0,n}}$$

Percent of inhibition for electrochemical assay is expressed in a similar way to Ellman's assay. The current *i* indexed i indicates inhibited blood and n, non-inhibited (intact) blood. Time scale was the same as for Ellman's assay − 1 minute. The principle behind the electrochemical assay is seen in following reaction scheme:

(3) $$\begin{array}{c} {\text{CH}}_{3}{\text{COSCH}}_{2}{\text{CH}}_{2}{\text{N}}^{+}{\left({\text{CH}}_{3}\right)}_{3}\overset{\text{cholinesterase}}{\to }{\text{HSCH}}_{2}{\text{CH}}_{2}{\text{N}}^{+}{\left({\text{CH}}_{3}\right)}_{3}+{\text{CH}}_{3}{\text{COO}}^{-}+{\text{H}}^{+}\\ 2{\text{HSCH}}_{2}{\text{CH}}_{2}{\text{N}}^{+}{\left({\text{CH}}_{3}\right)}_{3}\overset{+410\;\text{mV}}{\underset{-2({\text{e}}^{-}+{\text{H}}^{+})}{\to }}{\left({\text{CH}}_{3}\right)}_{3}{\text{N}}^{+}{\text{CH}}_{2}{\text{CH}}_{2}{\text{S}}^{-}{\text{SCH}}_{2}{\text{CH}}_{2}{\text{N}}^{+}{\left({\text{CH}}_{3}\right)}_{3} \end{array}$$

### 3.4. Mathematical operations with achieved data

The achieved experimental data were processed in Origin 6.1 (Northampton, MA, USA). The calibration curves were estmated by a suitable function according to optimal coefficients of determination. Comparison of Ellman's method and the electrochemical sensor was performed as a linear dependence. Limits of detection were estimated as a point on the calibration curve corresponding to 3x blank standard deviation.

## 4. Conclusions

An electrochemical sensor is proposed here as a usefule tool for diagnosis of poisoning caused by organophosphates or carbamates, exemplified by the highly toxic compound VX. There was good correlation between the electrochemical sensor and the standard photometric assay, although the electrochemical sensor provided a slightly better limit of intoxication detection. The anticipated economical impact due to the simplicity of the assay was mentioned in the Results and Discussion section. The electrochemical strip was used repeatedly in order to reduce costs over the measurement cycle. Total costs per one assay are considerably lower to those of the photometric assay. We may conclude with the opinion that the electrochemical sensor is more convenient based on costs per assay as well as the price of the device, analytical parameters, portability and speed. The whole device could be simply automated in the future. The study represents continuous effort to construct smart devices for routinely diagnosis and *in vitro* characterization of hazardous analytes [19 - 26].
